# Supplementary material for: Study on Structure Activity Relationship of Natural Flavonoids against Thrombin by Molecular Docking Virtual Screening Combined with Activity Evaluation In Vitro
Source: Molecules. 2020 Jan 20;25(2):422. doi: 10.3390/molecules25020422 (PMC7024217; doi:10.3390/molecules25020422)
Supplement: Supplementary file 1 [file molecules-25-00422-s001.pdf]

molecules

Study on Structure Activity Relationship of Natural Flavonoids against Thrombin by Molecular Docking Virtual Screening combined with Activity Evaluation in vitro

Xiaoyan Wang[1, 2 #], Zhen Yang[3, 4 #], Feifei Su[3, 4], Jin Li[1], Evans Owusu Boadi [1, 2], Yan-xu Chang[1, 2*], Hui Wang[3, 4*]

[1] Tianjin State Key Laboratory of Modern Chinese Medicine, Tianjin University of Traditional Chinese Medicine, Tianjin, 300193, China;
[2] Tianjin Key Laboratory of Phytochemistry and Pharmaceutical Analysis, Tianjin University of Traditional Chinese Medicine, Tianjin, 300193, China;
[3] Tianjin Key Laboratory of Chinese medicine Pharmacology, Tianjin University of Traditional Chinese Medicine, Tianjin, 300193, China
[4] College of Chinese Materia Medica, Tianjin University of Traditional Chinese Medicine, Tianjin, 300193, China

* Corresponding author: tgwanghui@163.com (H.W.); tcmcyx@tjutcm.edu.cn (Y.C)
Tel. /FAX: +86-22-59596163(S.F. &Y.C.)
# These authors contributed equally to this work.

Table S1 the docking score of 103 Compounds (In descending order of "-CDOCKER_energy")

Figure S1 the Molecular structures of 42 flavonoids

Table S1 the docking score of 103 Compounds (In descending order of "-CDOCKER_energy")

No.	CAS	compound	-CDOCKER_energy (kcal/mol)	-CDOCKER_INTERACTION_energy (kcal/mol)
1	1257-08-5	(-)-Epicatechin gallate	52.0751	54.6293
2	83104-87-4	(-)-EGCG-3"-O-Me	51.2391	50.5546
3	20315-25-7	Proanthocyanidin B1	42.3509	55.9285
4	27200-12-0	Dihydromyricetin	40.3383	43.3260
5	489-35-0	Gossypetin	37.3506	37.1098
6	529-44-2	Myricetin	36.234	37.7207
7	520-33-2	Hesperetin	35.4752	41.9244

8	520-26-3	hesperidin	35.4752	41.9244
9	529-53-3	Scutellarein	35.0756	35.6478
10	970-73-0	(+)-Gallocatechin	34.8439	40.9161
11	90-19-7	Rhamnetin	34.588	40.0848
12	480-18-2	dihydroquercetin	34.3549	39.6933
13	24211-30-1	Farrerol	34.1885	39.5947
14	520-18-3	Kaempferol	33.6353	40.2301
15	20243-59-8	3,5-dihydroxy-2-(4-hydroxyphenyl)-7-methoxy-4H-chromen-4-one	33.6121	38.5550
16	970-74-1	(-)-epigallocatechin	33.4333	39.2926
17	528-48-3	Fisetin	33.4140	36.2051
18	480-16-0	Morin hydrate	32.9997	39.3092
19	492-14-8	7,3,4-Trihydroxyflavanone	32.7988	35.309
20	6151-25-3	Quercetin dihydrate	32.7639	34.9759
21	17-39-5	Quercetin	32.2276	34.3347
22	916917-28-7	5,7,4'-Trihydroxy-8-methylfavnone	31.6178	36.6375
23	6674-40-4	5-hydroxy-7-acetoxylsaccharidesavone	31.5674	35.9231
24	490-46-0	L-Epicatechin	31.5442	36.5319
25	491-70-3	Luteolin	31.5122	36.5693
26	520-34-3	Diosmetin	31.4054	38.3832
27	520-11-6	2-(3,4-Dihydroxyphenyl)-5,7-dihydroxy-6-methoxy-4H-chromen-4-one	31.0184	38.737
28	527-95-7	Herbacetin	30.9927	31.1996
29	480-41-1	Naringenin	30.3078	35.498
30	18422-83-8	Dihydromorin	30.2329	37.3532
31	480-20-6	Dihydrokaempferol	29.9901	36.438
32	480-43-3	Isosakuranetin	29.5562	37.5935
33	1447-88-7	Hispidulin	29.4917	38.2314
34	51059-44-0	Wogonoside	29.4917	38.2314
35	29424-96-2	(S)-5-Hydroxy-7-methoxy-2-(4-methoxyphenyl)chroman-4-one	29.3475	39.2539
36	480-19-3	Isorhamnetin	29.2294	35.0669
37	2957-21-3	Sakuranetin	29.0545	36.6535

38	520-12-7	Pectolinarigenin;Pectolinaringenin	29.0249	41.4103
39	2196-14-7	4H-1-Benzopyran-4-one,7-hydroxy-2-(4-hydroxyphenyl)-	28.9851	33.9775
40	491-67-8	Baicalein	28.6997	28.4595
41	7295-85-4	catechin	28.3311	35.6321
42	480-44-4	5,7-Dihydroxy-4'-methoxyflavone	28.2615	35.5928
43	18085-97-7	Jaceosidin	27.9437	42.9346
44	548-83-4	Galangin	27.5273	32.1012
45	5128-44-9	4',7-Dimethoxy-5-Hydroxyflavone	27.4778	37.943
46	14965-20-9	3,6,7-Trimethylquercetagetin	26.8000	45.0216
47	1621-55-2	3-Hydroxyflavone	26.2056	30.6068
48	437-64-9	Genkwanin	26.0983	33.2256
49	520-36-5	Apigenin	25.8594	30.7509
50	480-40-0	chrysin	25.4937	30.3195
51	22688-79-5	Quercetin 3-O-β-D-Glucuronide	24.7850	48.7562
52	28608-75-5	Orientin	24.7432	45.7606
53	17313-52-9	Centaureidin	23.6831	46.1836
54	16290-07-6	Kaempferol-7-O-β-D-glucopyranoside	22.6377	48.5403
55	78417-26-2	5,6,3'-Trihydroxy-6,4',5'-triMethoxyflavo ne	22.6090	39.6538
56	18956-18-8	Dihydrooroxylin	22.5692	31.7716
57	22368-21-4	Eupatilin	22.2295	44.2855
58	4143-62-8	3',4'-Dimethoxyflavone	21.2458	33.3833
59	2798-20-1	Gardenin B	20.8488	45.5232
60	603-56-5	chrgsosplenetin B	20.5357	44.4268
61	21967-41-9	Baicalin	20.4921	40.0299
62	632-85-9	Wogonin	20.0775	28.6305
63	25739-41-7	Velutin	19.9638	40.37
64	861691-37-4	2''-O-beta-L-galactopyranosylorientin	19.8823	58.3188
65	491-50-9	4H-1-Benzopyran-4-one,2-(3,4-dihydroxyphenyl)-7-(b-D-glucopyranosyloxy)-3,5-dihydroxy	19.5093	46.3709
66	86450-80-8	Sanggenone H	19.3726	37.518
67	652-78-8	GOSSYPIN	18.7432	46.6315

68	528-53-0	Delphinidin	17.8924	36.6785
69	6601-66-7	6-Demethoxytangeretin	17.5812	38.0676
70	522-12-3	Quercitrin	17.5434	51.1847
71	604-59-1	Alpha-Naphthoflavone	17.4933	32.148
72	17912-87-7	Myricetrin	17.3745	50.2632
73	740-33-0	Mosloflavone	17.2521	32.3742
74	549-32-6	REYNOUTRIN	17.2345	43.2093
75	5240-95-9	Icaritin	16.869	45.0692
76	527-95-7	Herbacetin	16.3798	47.6805
77	112448-39-2	2-(3-hydroxy-4-methoxyphenyl)-5,6,7,8-tetramethoxychromen-4-one	16.1159	48.9596
78	53846-50-7	8-PRENYLNARINGENIN	15.1227	42.0417
79	5041-82-7	Isorhamnetin-3-O-glucoside	14.8513	54.9687
80	158196-34-0	Naringenin-7-O-glucuronide	14.8472	44.4411
81	13306-05-3	anthocyanidins	14.6415	35.461
82	6991/10/2	APIGENIN6-GLUCOSYL-7-O-METHYLETHER	14.4213	42.8616
83	5373/11/5	Luteoloside	14.0802	45.435
84	35775-49-6	Chrysin 7-glucuronide	13.899	41.1444
85	482-39-3	4H-1-Benzopyran-4-one,3-[(6-deoxy-a-L-mannopyranosyl)oxy]-5,7-dihydroxy-2-(4-hydroxyphenyl)-	13.7985	45.5269
86	529-55-5	Naringenin-7-O-β-D-glucoside	13.7947	48.7942
87	29702-25-8	Isovitexin	13.6155	36.9486
88	482-35-9	Quercetin-3-O-β-D-glucoside	13.5913	58.8274
89	479-90-3	ARTEMETIN(6CI)	13.1101	41.4924
90	482-36-0	Hyperoside	12.6667	46.9391
91	19879-30-2	BAVACHININ A	12.6085	44.7526
92	19879-32-4	BAVACHIN	12.5302	40.4062
93	578-74-5	Apigenin-7-O-β-D-glucopyranoside	11.7529	45.2264
94	1251-84-9	Quercetagetin 3,5,6,7,3',4'-hexamethyl ether	10.937	53.5872
95	14259-46-2	Narirutin	10.8038	55.6454
96	480-10-4	Kaempferol-3-O-glucoside	7.92061	46.7529

97	478-01-3	Nobiletin	7.874	46.183
98	10236-47-2	Naringin	7.64425	56.2504
99	13241-33-3	neohesperidin	6.28391	60.1197
100	55033-90-4	Isorhamnetin-3-O-neohespeidoside	5.76395	57.2055
101	153-18-4	Rutin	5.75821	61.7163
102	17650-84-9	Kaempferol 3-rutinoside	3.26751	58.4229
103	104472-68-6	Typhaneoside	2.96544	75.8091

molecules

Figure S1 the Molecular structures of 42 flavonoids

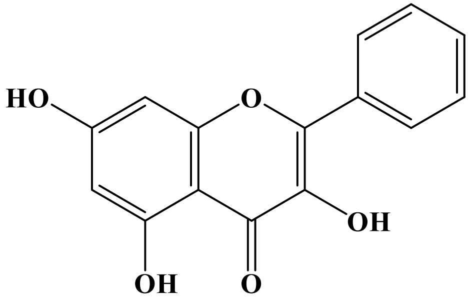

1.Galangin

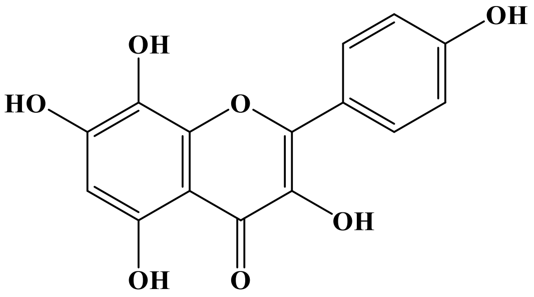

2.Herbacetin

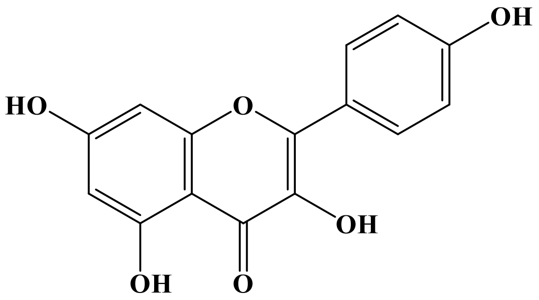

3.Kaemperol

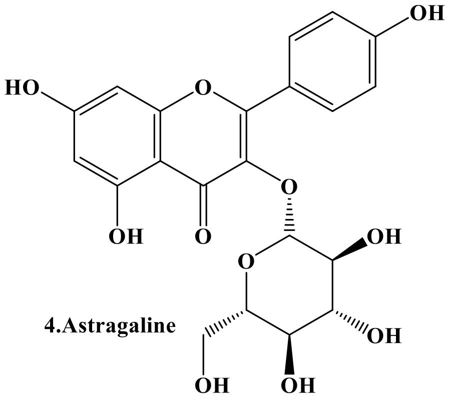

4.Astragaline

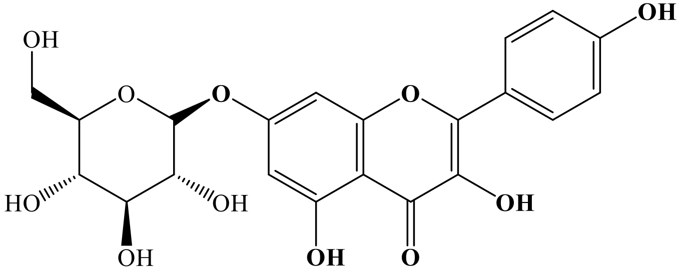

5.Kaempferol-7-O-β-D-glucopyranoside

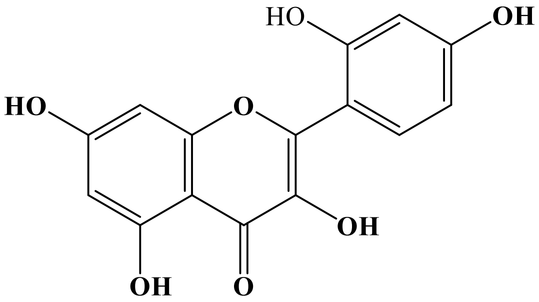

6.Morin

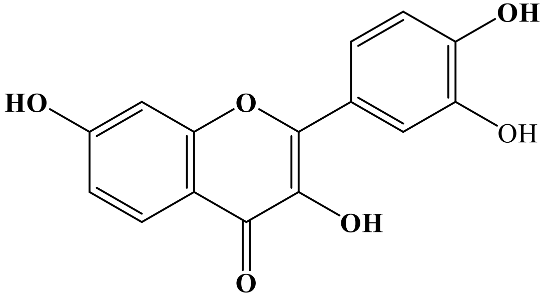

7.Fisetin

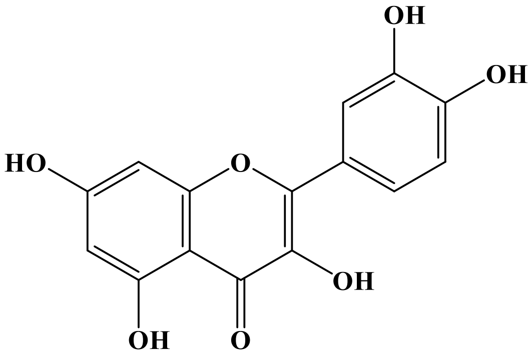

8.Quercetin

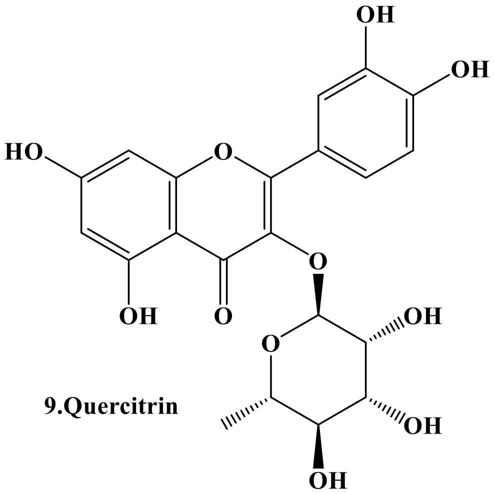

9.Quercitrin

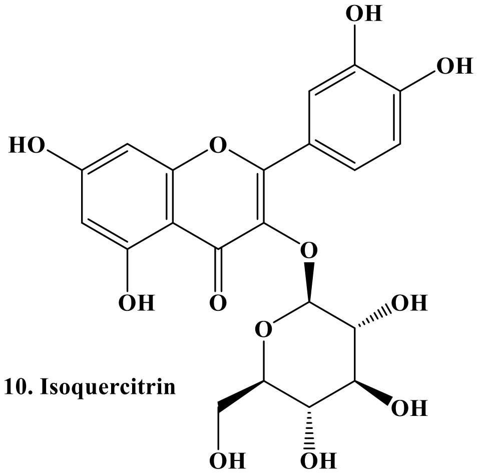

10. Isoquercitrin

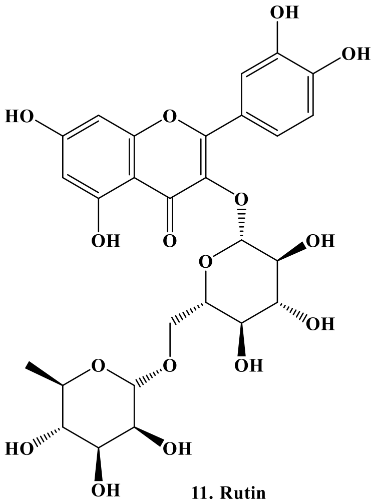

11. Rutin

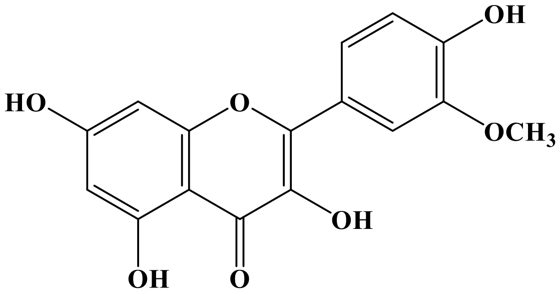

12. Isorhamnetin

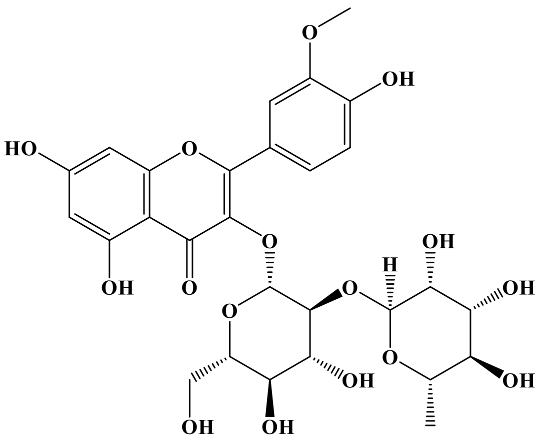

13. Isorhamnetin-3-O-neohespeidoside

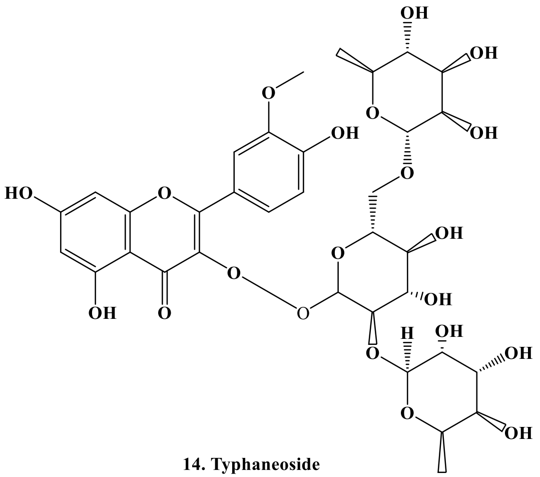

14. Typhaneoside

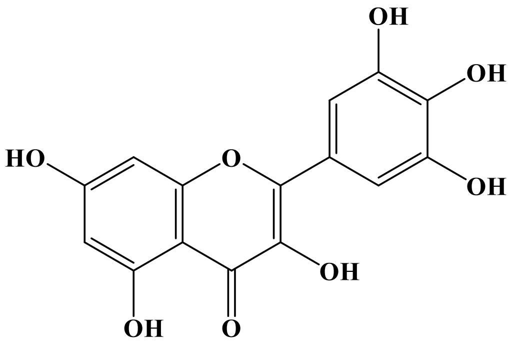

15. Myricetin

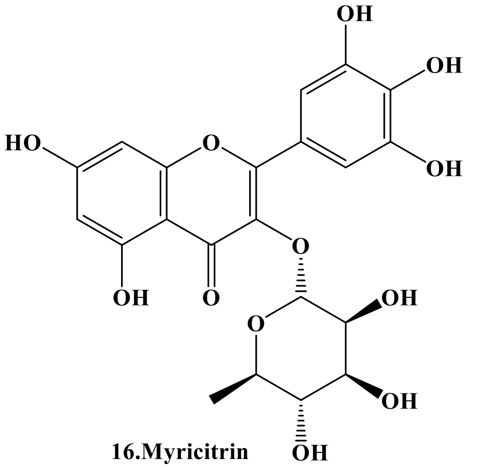

16.Myricitrin

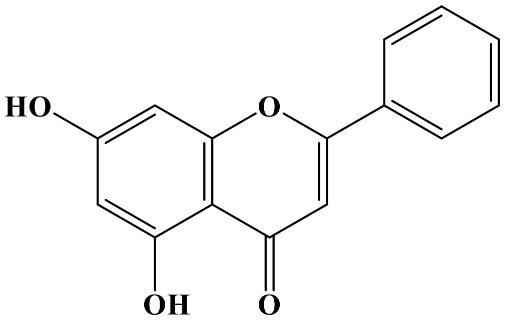

17. Chrysin

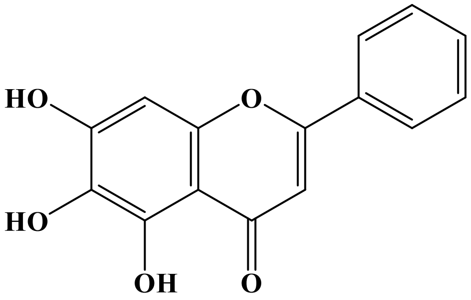

18. Baicalein

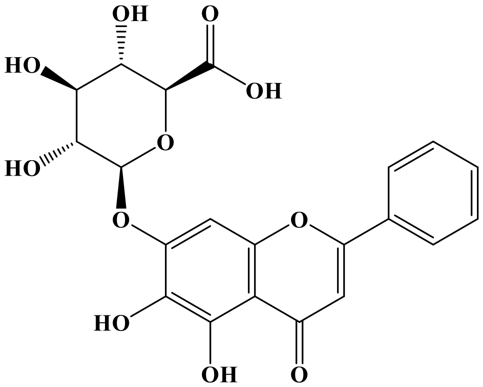

19. Baicalin

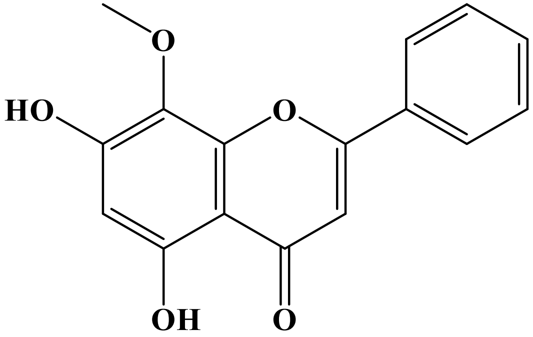

20. Wogonin

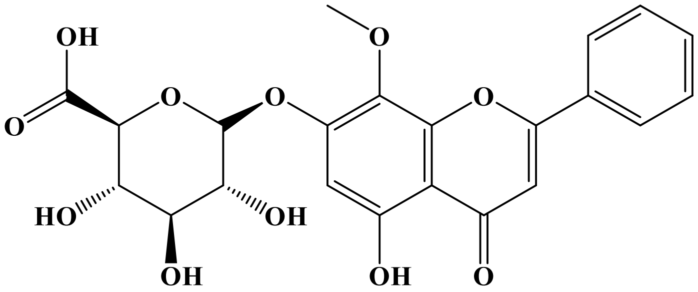

21. Wogonoside

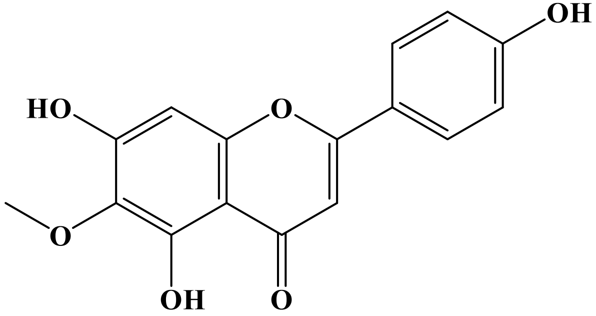

22. Hispidulin

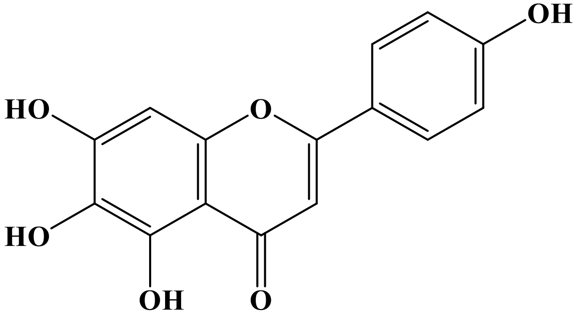

23. Scutellarein

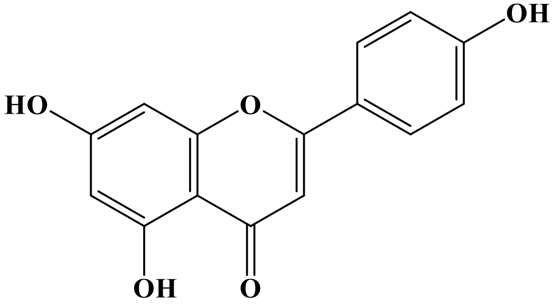

24. Apigenin

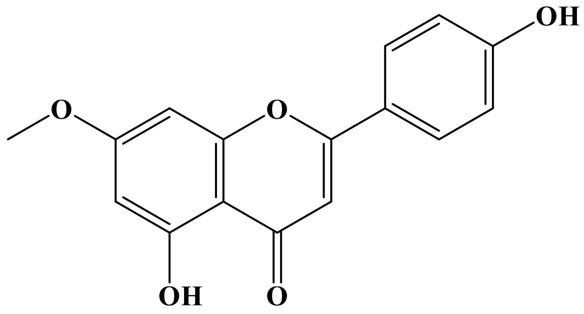

25. Genkwanin

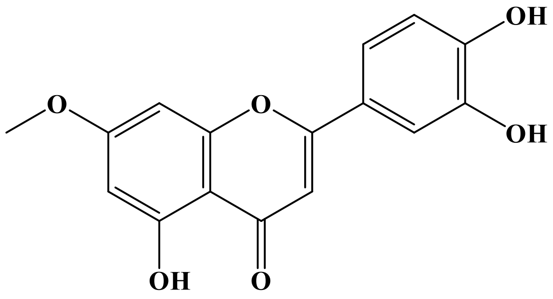

26. Hydroxygenkwanin

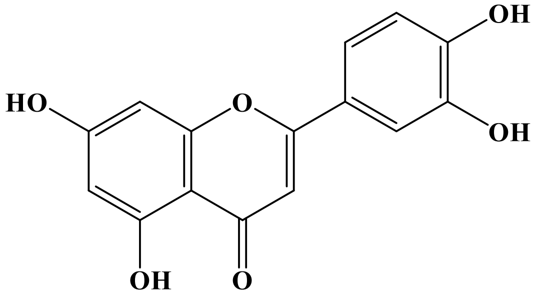

27. luteolin

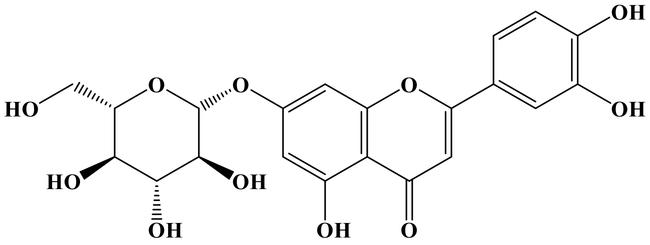

28. Luteoloside

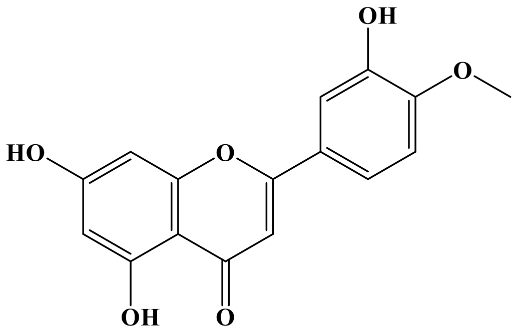

29. Diosmetin

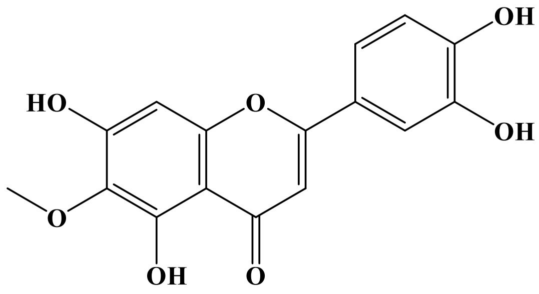

30. 6-Methoxyluteolin

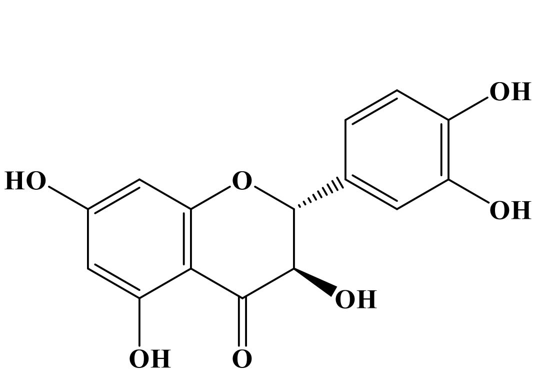

31. Dihydroquercetin

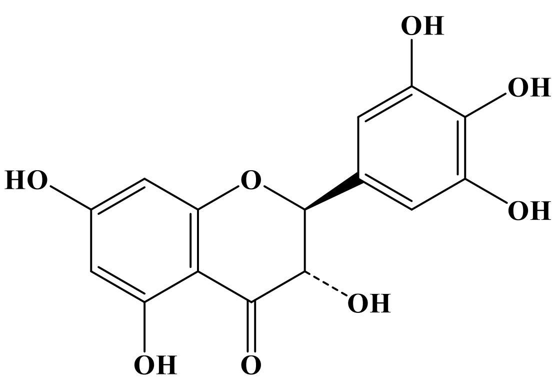

32. Dihydromyricetin

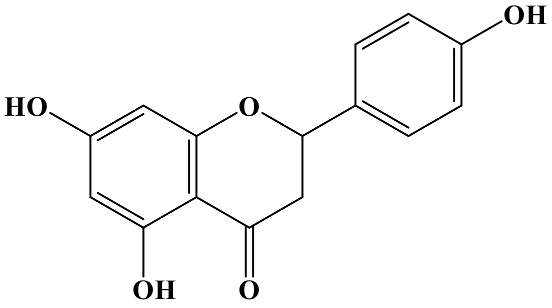

33. Naringenin

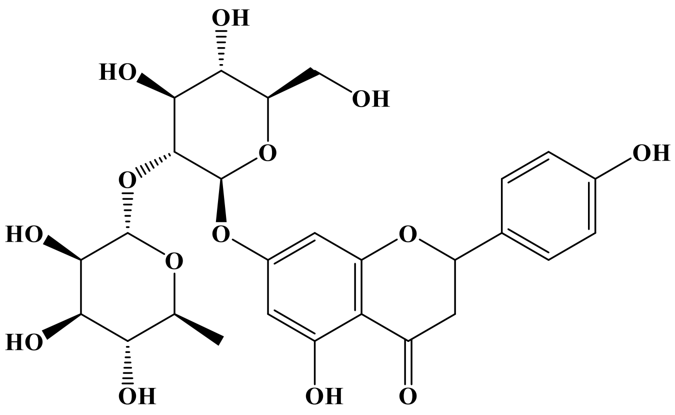

34. Naringin

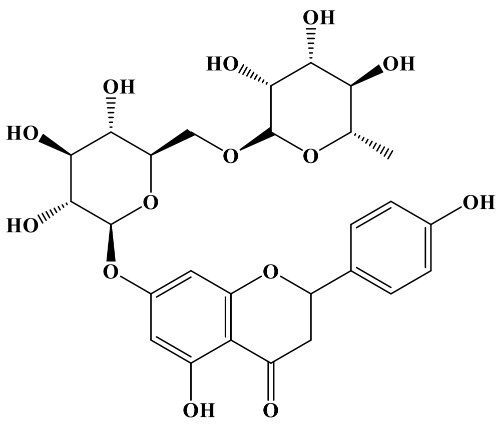

35. Narirutin

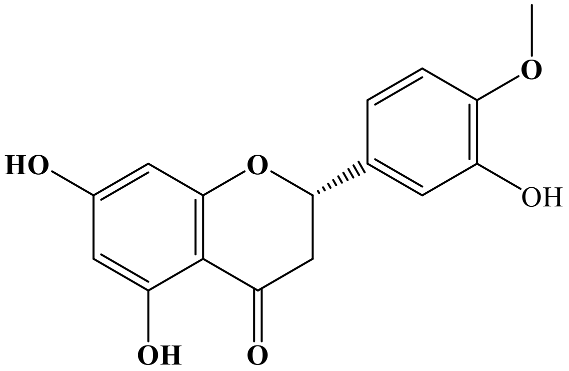

36. Hesperetin

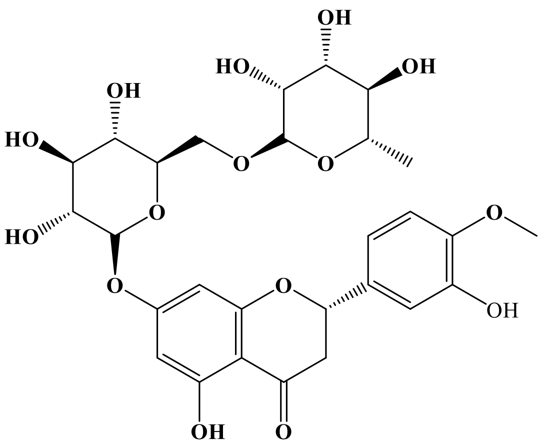

37. Hesperidin

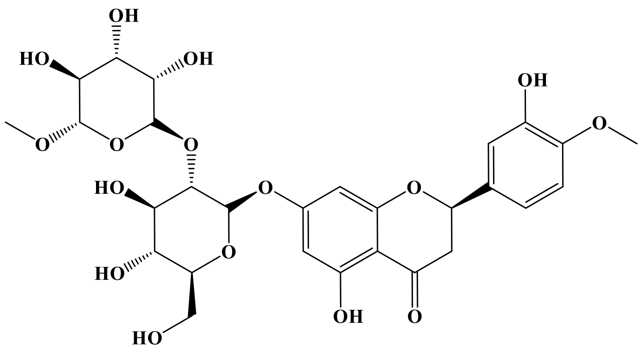

38. Neohesperidin

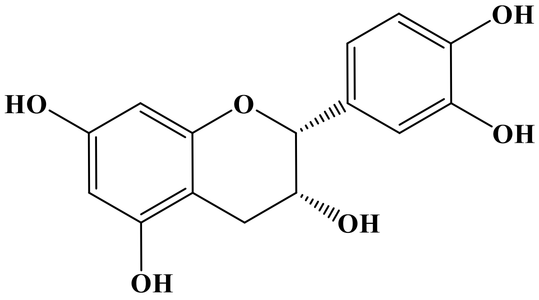

39. Epicatechin

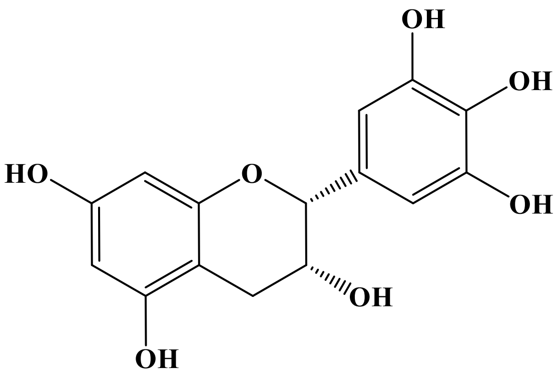

40. (-)-epigallocatechin

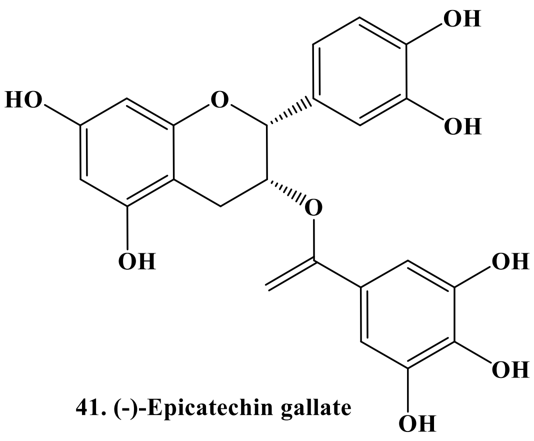

41. (-)-Epicatechin gallate

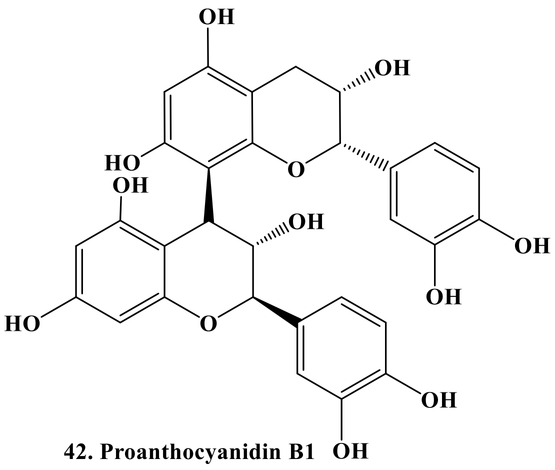

42. Proanthocyanidin B1
